# A novel computational model for human macular pigment optical density and its relationship to foveal structure

**DOI:** 10.1038/s41598-025-21681-4

**Published:** 2025-10-29

**Authors:** Gary P. Misson, Stephen J. Anderson, Richard A. Armstrong, Rebekka Heitmar

**Affiliations:** 1 School of Optometry, College of Health and Life Sciences, Aston University, Birmingham, UK; 2 South Warwickshire NHS Foundation Trust, Lakin Road, Warwick, UK; 3 Centre for Vision across the Life Span (CVLS), School of Applied Sciences, University of Huddersfield, Huddersfield, UK

**Keywords:** Computational models, Retina, Retina

## Abstract

Macular pigment optical density (MPOD) models enhance understanding of macular xanthophyll distribution, particularly relevant to age-related macular degeneration. This study investigates an existing model and introduces a novel, more accurate and biologically relevant approach. MPOD spatial profiles of 48 eyes were obtained using dual-wavelength autofluorescence imaging, with structural data from OCT and OCT-angiography. MPOD data were analyzed using (a) an existing sum of exponential and Gaussian model (M_EG_) and (b) a novel sum of three Gaussians model (M_3G_). Extracted parameters generated individualized MPOD models, from which gradients and volumes were derived. M_3G_-derived variables were analyzed against OCT/OCTA data using factor analysis and multiple regression. M_3G_ demonstrated a superior fit to MPOD data (SSE = 2.60 × 10^− 3^) compared to M_EG_, (SSE = 35.7 × 10^− 3^) enabling automated fitting consistent over small and large datasets. M_3G_ provided meaningful variables, including MPOD gradients, volumes and critical point eccentricities. Correlations included those between dependent variables of critical point eccentricities and central macular pigment volume with foveal avascular zone and foveal pit radii.

The excellent data fit of M_3G_ enables automated extraction of physiologically relevant parameters. Its three-component configuration is consistent with the location of macular xanthophylls. M_3G_ is similar to models of foveal structure, suggesting a fundamental relationship.

**Supplementary Information:**

The online version contains supplementary material available at 10.1038/s41598-025-21681-4.

## Introduction

The plant-derived yellow xanthophyll carotenoids, lutein and zeaxanthin, along with the metabolite mesozeaxanthin, are concentrated in the anatomically specialised region of the retina known as the macula lutea (Latin for ‘yellow spot’, commonly abbreviated as the ‘macula’)^1,2^. Collectively referred to as macular pigment (MP), these compounds are thought to optimise visual function, protect against short-wavelength visible light and scavenge free radicals^3,4^. It is now accepted that MP plays a protective role in reducing the risk of age-related macular degeneration (AMD)^5^: MP deficiency increases the risk of AMD^6^, while MP supplementation can mitigate this risk^7^.

Dual-wavelength autoflourescence^8^ has emerged as a reliable and accurate objective method for measuring in vivo macular pigment optical density (MPOD), a quantifier of macular pigment density^9^. MPOD data is typically plotted against circumferential eccentricity from a point centred on the foveola, creating a macular pigment spatial density profile^9^ .

Macular pigments are concentrated in the central fovea, declining to < 10% of peak value by 3° eccentricity (approximately 1 mm from the foveolar centre) and stabilizing at a low, constant level from about 7° eccentricity. Zeaxanthin and its isomer, meso-zeaxanthin, are confined to the fovea^2^. It is particularly concentrated in the inner plexiform layer, outer plexiform layer and outer nuclear layer of the fovea and foveola, most likely in association with the Mueller cell cone^10,11^. This supports the hypothesis that Mueller cells, rather than photoreceptor axons^12^, serve as the principal macular pigment reservoir^10,11,13,14^. Lutein, in contrast, is distributed more diffusely, located eccentrically to the fovea and at lower concentrations^2,11,15^.

There are considerable inter-individual variations in the spatial distribution of macular pigments, leading to qualitative spatial profile descriptions such as ‘central peak’, ‘ring-like’, ‘central dip’ and ‘intermediate’^16^. Some spatial density profiles exhibit a strictly monotonic decline with eccentricity from a central peak. Other profiles, while still declining monotonically, display gradient changes that form an eccentric shoulder. Non-monotonic profiles, on the other hand, feature eccentric peaks in the presence or absence of a central peak. The latter represent an annular or ‘ring-like’ pattern in the two-dimensional MPOD profile^17^.

Variations in MPOD spatial profiles are linked to morphological differences. For example, the presence of eccentric peaks in MPOD spatial profiles are associated with wider foveae^18^, and eyes with larger foveal avascular zones (FAZ) are more likely to exhibit an eccentric peak in their MPOD spatial profiles^19^.

Despite the variation in MP spatial profiles, these have been represented using computational models, including simple exponential^20^, Gaussian^21,22^, combined exponential-Gaussian^23^ and a two-dimensional Zernike polynomial model^24^. These models were selected to provide the ‘best fit’ to the data rather than being guided by physiological or anatomical principles. They have proven useful for classifying macular pigment profile variations^23^ and predicting central macular pigment optical density from noisy datasets^22^.

Analytic models enable complex forms, such as the variety of macular pigment spatial profiles, to be represented accurately using a small set of parameters. The parameters themselves can be analysed and used to reconstruct individual profiles with greater precision, allowing for more detailed analyses than may be possible with experimental data alone. Such models also serve as a foundation for the automatic extraction of quantitative features with potential clinical utility, such as peak values, volumes and rates of attenuation. Furthermore, accurate models can provide deeper insights than numeric data alone, as has shown to be the case for retinal morphology defined by optical coherence tomography (OCT), (see, for example^25,26^.

The present study aimed to develop and test an accurate analytic model of the macular pigment optical density (MPOD) spatial profile. Initially, the Berendschot and van Norren model^23^ was selected for evaluation using dual-wavelength autofluorescence data obtained through now-standard methodologies^9^. This model was chosen because it consistently fit MPOD data and describes the retinal spatial profile of macular pigment as a continuous function. Such a function enables the derivation of properties such as gradients and changes in gradients through differentiation and volumetric data through integration, although such analyses have not yet been reported in the literature for MPOD.

However, preliminary analyses revealed that the Berendschot and van Norren model did not fully capture the complexity of the experimental data. These findings indicated the need for a more nuanced approach, leading to the proposal of a three-component model. Each component of this novel model represents a specific region of the MPOD spatial profile, accounting for variations not addressed by the original two-component model.

The study then shifted its focus to developing, verifying and interrogating this three-component model. The model was rigorously tested against measured MPOD data and structural data obtained through optical coherence tomography (OCT) and OCT angiography (OCTA).

The new model successfully represents experimental data with high accuracy, correlates with structural data and generates novel parameters that represent the location and concentration of macular pigment in the human retina. These findings demonstrate the model’s potential for advancing both research and clinical applications related to macular pigment and retinal health.

**Fig. 1:**
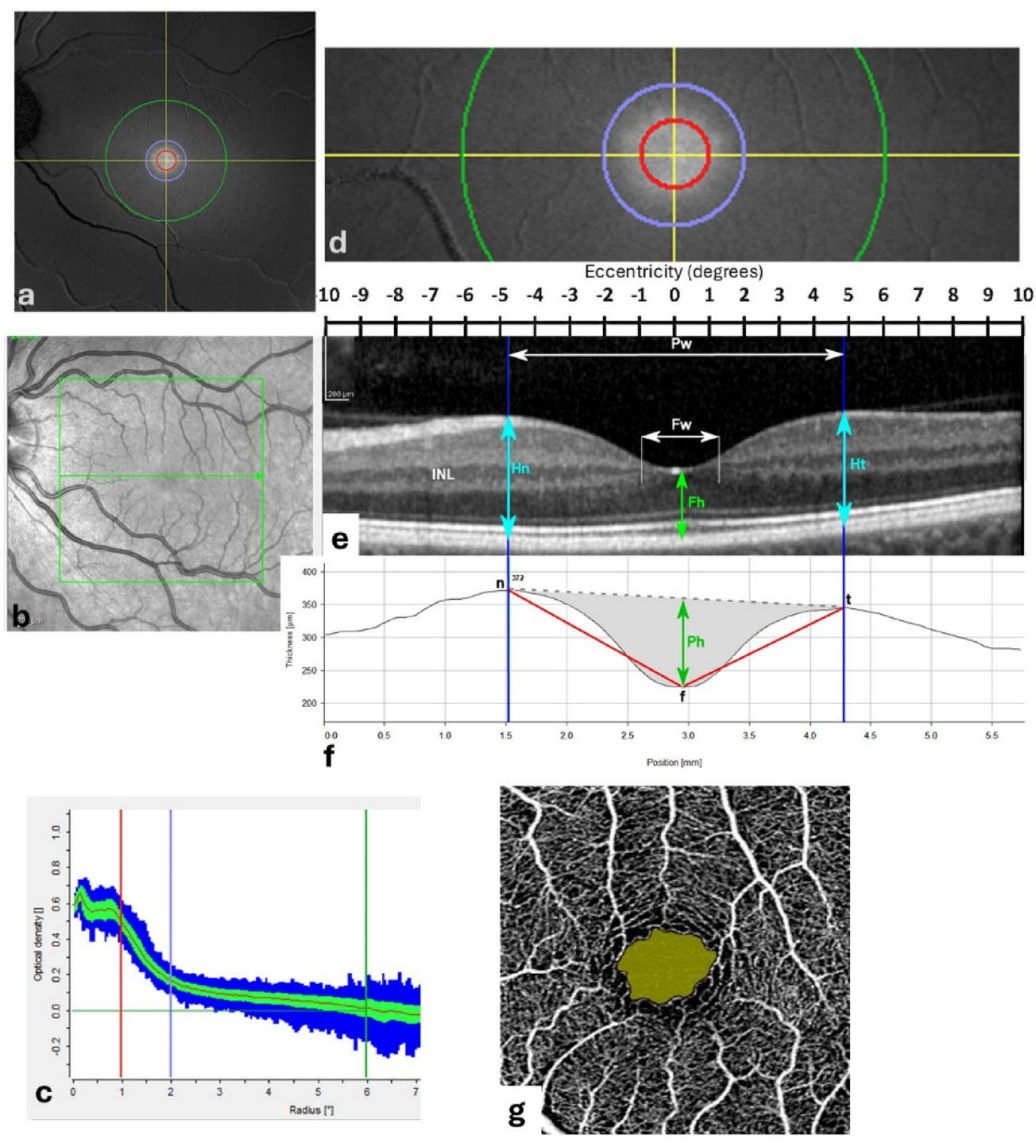
Fundal imagery and definitions: Case # 41. (**a**) Dual wavelength autoflourescence image of the left macular region, with circles marking eccentricities of 1° (red), 2° (blue) and 6° (green). (**b**) Infrared scanning laser ophthalmoscopy (SLO) fundus image of the same field as in (**a**), with 20°x 20° box (green) superimposed. (**c**) Machine-generated plot showing the mean and standard deviation of macular pigment optical density (MPOD) measured along circular paths at increasing eccentricities: red, blue and green vertical lines mark the 1°, 2° and 6° circles shown in (**a**). (**d**) Enlarged 20° x 6° dual-wavelength autoflourescence image across the fovea along the green arrow in (**b**). (**e**) Optical coherence tomography (OCT) image of the fovea along the green arrow in (**b**). (**f**) Machine-generated retinal thickness profile (distance between retinal pigment epithelium (RPE) and inner limiting membrane (ILM)) with machine calibration values. (**g**) 10° x 10° OCT angiography (OCTA) image showing machine-defined foveal avascular zone (FAZ). Annotations: INL, inner nuclear layer of retina; f, centre of foveola; Fh, foveolar height (light green); Fw, foveolar width (white); n/t, nasal/temporal peak macular thickness (light blue); Pw, foveal pit width; Ph, foveal pit height (dark green). The foveal angle is angle between lines drawn between n to f, and f to t (**f**).

## Experimental methods and data collection

Data collection was conducted at the School of Optometry at Aston University, UK, between January and December 2021. The study adhered to the tenets of the Declaration of Helsinki and was approved by Aston University Local Ethics Committee (application #1566). All participants gave informed consent to participate in the study. Exclusion criteria were prior history or clinical evidence of ocular disease. Measurements were taken from 48 eyes, drawn from a participant pool of 13 males and 12 females (age range: 28 to 64 years; mean age ± standard deviation: 46.2 ± 11.7 years). All participants were white Europeans with the exception of two male participants who were British Asian. Whilst variations in macular pigment distribution occur in different ethnic groups^9,27^, the ethnicity of participants was not relevant to the aims and outcomes of the present study.

OCT and MPOD measurements (Table 1) were obtained using the Heidelberg *Spectralis* OCT/SLO (Heidelberg, Engineering, Heidelberg, Germany) following the manufacturer’s instructions and protocols detailed in previous studies^9,14,28,29^. The dual-wavelength autoflourescence module was used to extract macular pigment data (Fig. 1a, c, d), including averaged MPOD values and MPOD volume measures along and within circular paths with radii extending from the foveal centre of 0.2°, 1°, 2° and a reference radius of 6° (corresponding to machine calibrated values of 0.20°, 0.98°, 1.99° and 5.98°).

Additional data extraction from the blue and green autofluorescence images was performed using *ImageJ* image analysis software^30^ and analysed following the methods of Kar et al.^14^ using their open source software (https://sites.imagej.net/CreativeComputation/).

Spectral-domain OCT macular images were presented as 20° x 20° blocks, with a single horizontal scan passing through the foveolar centre (Fig. 1b, e). Morphometry was performed using *ImageJ*. Morphometric landmarks, parameters and values are summarised in Table 1; Fig. 1e, f. The foveolar radius (Fr) was defined as half the distance between adjacent terminations of the inner nuclear layer (INL) in the horizontal plane of the OCT scan (INL and Fw in Fig. 1e). The foveal pit radius (Pr) was defined as half the distance between the maximum nasal and maximum temporal macular thicknesses in the horizontal plane OCT (Pw, n, t in Fig. 1e).

Degrees of visual angle were used for linear measurements in OCT and OCT angiography images to ensure consistency across different measurements and to avoid confounding errors due to magnification effects from inter-individual variations in ocular dimensions^31^. Machine-generated axial measures of retinal thicknesses were expressed in micrometers (µm).

OCT angiography was performed using the Cirrus 5000 HD-OCT (Carl Zeiss Meditec Inc., Dublin, CA, USA. software version 11.0.0.29946). Machine-generated values and foveal avascular zone (FAZ) delineation were obtained using the proprietary software with the 3 mm x 3 mm angiography setting (Fig. 1g). Images of machine delineated FAZ were further analysed and measured using *ImageJ*. Morphometric results were again expressed in degrees of visual angle, with the examination field assumed to be constant at 10° x 10°. Three FAZ metrics were recorded (Table 1): the maximum Feret’s (calliper) diameter (FAZf), the mean horizontal and vertical diameters (FAZm) and the equivalent radius (FAZer), calculated as the radius of a circle with the same area as the FAZ^19^.

**Table 1:** Data definitions and summary of macular pigment optical density (MPOD), OCT and OCTA measures for the 48 eyes. 95% CI is the 95% confidence Interval.

	Macular pigment optical density (arbitrary units)	Mean	Min	Max	sd	95%CI
MPOD						
OD rad0.2	At 0.2° eccentricity	0.59	0.25	1.03	0.20	0.53–0.64
OD rad 1	At 1° eccentricity	0.37	0.15	0.67	0.14	0.33–0.41
OD rad 2	At 2° eccentricity	0.12	0.03	0.22	0.05	0.11–0.14
OD rad 3	At 3° eccentricity	0.01	0.00	0.02	0.01	0.01–0.01
sumV0.2	Volume to 0.2° eccentricity	57	25	101	19	52–63
sumV 1	Volume to 1° eccentricity	924	408	1629	333	829 1018
sumV 2	Volume to 2° eccentricity	2184	846	3843	828	1950–2418
sumV 3	Volume to 3° eccentricity	5108	1581	9500	1999	4542–5674
Peak	Central peak	0.64	0.28	1.10	0.20	0.58–0.69
OCT						
Av CRT	Average central retinal thickness (µm)	274	246	299	14	270–278
Fr	Foveolar radius (degrees)	0.81	0.51	1.13	0.16	0.77–0.86
Pr	Foveal pit radius (degrees)	3.96	2.80	5.09	0.44	3.83–4.08
fov ang	Foveal pit angle (degrees)	168	160	172	3	167–169
fov th	Central foveal thickness (µm)	231	206	272	14	227–235
bwlht	Foveal bowl height (µm)	123	68	166	22	117–129
OCTA						
FAZf	Foveal avascular zone maximum Feret’s diameter (degrees)	2.27	1.3	2.88	0.41	2.15–2.39
FAZm	Foveal avascular zone mean diameter (height + width)/2 (degrees)	2.03	1.01	2.60	0.39	1.92–2.14
FAZer	Foveal avascular zone equivalent radius $\left( {\sqrt {FAZ\;Area/\pi } } \right)$ (degrees)	0.91	0.40	1.17	0.19	0.85–0.96

### Part 1: the existing model

#### Analysis and modelling

Mathematical manipulation, data processing and non-linear regression analyses were performed using Wolfram *Mathematica* (Wolfram Research, 2024; Version 11.1.1.0). Nonlinear regression was initially performed between MPOD data and the two-component model of Berendschot van Norren^23^. This model (M_EG_, Eq. 1) represents the sum of a declining exponential component and a Gaussian component: the exponential component accounts for the central MPOD peak and peripheral tail-off, while the Gaussian component accounts for any eccentric shoulders or eccentric peaks. Originally expressed in base 10, the Berendschot van Norren model is here expressed in base ***e*** for consistency and mathematical simplicity, and is defined as:

1 $$\:{\text{M}}_{\text{E}\text{G}}(\text{x};{\text{A}}_{1},{\text{p}}_{1},{\text{A}}_{2},{\text{p}}_{2},{\text{x}}_{2})=\:{\text{A}}_{1}.{e}^{-{\text{p}}_{1}.x}+{\text{A}}_{2}.{e}^{-{\text{p}}_{2}.{\left(x-{\text{x}}_{2}\right)}^{2}}$$

where the primary parameters A_1_ is the exponential amplitude, p_1_ the exponential decay constant, A_2_ the Gaussian amplitude, p_2_ the spread (‘peakedness’) of the Gaussian component, and x_2_ the eccentricity of the Gaussian component peak. Conversion to the base 10 model requires division of A_1_ and A_2_ by log_e_10, while all other parameters remain unchanged.

### Results 1 M_EG_ data fitting

The M_EG_ model was fitted to DWAF-derived MPOD profile data from 0° to 2° and 0° to 5° eccentricity using *Mathematica’s* non-linear data fitting algorithms for each of the 48 eyes and for the mean of the 48 eye MPOD data set as a function of eccentricity (the MPOD grand mean). Data fitting was performed both automatically and with manual control of parameters. The deviation between model-predicted and experimental data was quantified as the sum of squared differences (Table 2).

**Fig. 2:**
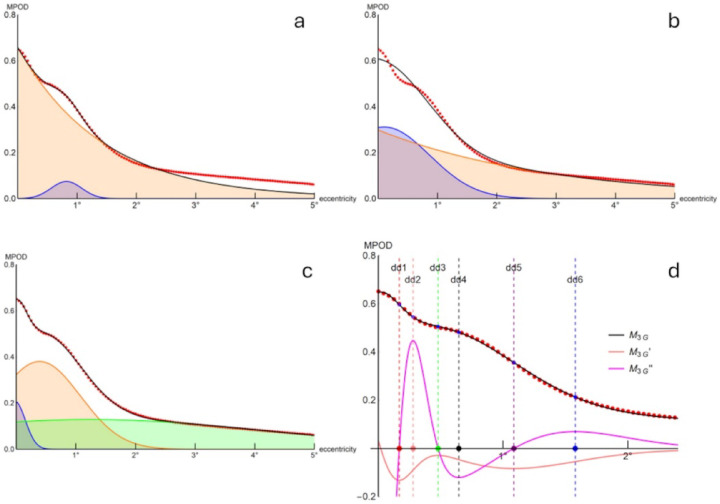
Grand mean of MPOD data (red dots) with model fit (black continuous line), model components (shaded areas and coloured continuous lines) and derivatives (coloured continuous lines in **d**). (**a**) M_EG_ model with data fit from 0° to 2° and (**b**) from 0° to 5° eccentricity, with separation into exponential (orange line/shade) and Gaussian (blue line/shade) components. (**c**) M_3G_ model with data fit from 0° to 5° eccentricity, with separation of Gaussian components (i = 1, blue; i = 2 orange; i = 3, light green). (**d**) as (**c**) for 0° – 2.5° eccentricity, showing first (orange line) and second (magenta line) derivatives with critical points dd_1_ – dd_6_ (coloured circles on x-axis with corresponding vertical dashed lines). See text for details.

The M_EG_ model fitted the 2° data set (Fig. 2a) with less deviation from the data than the 5° data set (Fig. 2b). When applied to the 5°data set, the model prioritized overall fit at the expense of finer details, such as the eccentric shoulder (Fig. 2b). In most cases, the 2° data set fit captured any eccentric peak or shoulder in the MPOD distribution (Fig. 2a) however did not offer an adequate fit when extended to the full 5° data set.

Manual adjustment of primary parameters to optimize the fit for the central peak and tail-off came at the expense of accurately capturing the eccentric shoulder or peak. Similarly, manipulation of primary parameters to best fit the eccentric shoulder/peak was at the expense of the central peak and peripheral tail-off (compare Fig. 2a and b).

The incomplete fit led to the conclusion that the M_EG_ model was not sufficiently accurate to represent the full extent of the macular pigment optical density profile, warranting the exploration of alternative models. Furthermore, the inability of the two-component model to simultaneously capture the central peak and peripheral tail-off while preserving any intermediate shoulder or peak suggests that a three-component model may provide a better fit.

**Table 2:** Comparison of data fits for the M_EG_ and M_3G_ models. Summed squared of difference between data and model predicted values for the 48 eyes. The data fit is either to 2° or 5°. Parameters from the 2° data fit were then applied to the 2° and 5° data sets separately (M_EG_ model: second and third results rows; M_EG_ model: fifth and sixth rows; 95% CI is the 95% confidence Interval).

Model	Fit to	Data to	Summed squares of difference between data and model x10^− 3^				
			Mean	Min	Max	Sd	95%CI
M_EG_	2°	2°	11.0	0.8	61.8	16.5	6.3–15.6
	2°	5°	118.8	8.7	333.6	83.7	95.1 -142.5
	5°	5°	35.7	1.6	109.3	24.8	28.6–42.7
M_3G_	2°	2°	1.4	0.2	8.3	1.4	1.0 -1.8
	2°	5°	641.4	2.4	13212.6	2083.3	52.0–1230.8
	5°	5°	2.6	0.5	8.3	1.7	2.1–3.1

### Part 2: A novel three-component Gaussian (M_3G_) model

Following trials of several three-component models, a three-Gaussian-component model (M_3G_ model; Eq. 2, Fig. 2c and d) provided the best fit to the data and was investigated further. The model,

2 $$\:{\text{M}}_{3\text{G}}(\text{x};{\text{N}}_{\text{i}},{\text{q}}_{\text{i}}\:,{g}_{i})=\sum\:_{i=1}^{i=3}{\text{N}}_{\text{i}}{e}^{-{\text{q}}_{\text{i}}{(x-{g}_{i})}^{2}}$$

is defined by the primary parameters N_i_, amplitude (scale parameter), g_i_ peak location (eccentricity of component peak); q_i_ the spread of components i = 1, 2, 3. Primary parameter ranges were constrained such that component i = 1 represented the central peak; i = 2 represented any intermediate eccentricity shoulders or eccentric; and i = 3 defined the peripheral tail-off.

For eccentricities x within physiological ranges, the function M_3G_ (as is M_EG_) is differentiable, with its first and second derivatives providing gradients and changes in gradients. These derivatives indicate mathematically definable critical points (e.g., inflections, turning points) that will be used in further analysis (Fig. 2d, Fig. 4).

The behaviour of the first derivative (M_EG_’, Fig. 2d) indicated whether M_EG_ was monotonically declining (M_EG_’ everywhere negative for the interval of interest) or nonmonotonic (M_EG_’ not everywhere negative). The eccentricities of the zeros, local minima and local maxima of the second derivative (M_EG_’’), when present, defined up to six quantifiable points of interest (derived parameters) in the models (Fig. 2d, Fig. 4). Each of these points represents a critical change in the gradient of the M_EG_ function. For example, the radius of the eccentric local minimum (dd_4_) represented the eccentricity of an eccentric peak or ‘shoulder’ of the MPOD spatial profile.

Five additional parameters of interest were derived from the M_EG_ model (see Supplementary Material for derivations): the central peak amplitude at zero degrees eccentricity A0_3G_ = M_3G_(0); the amplitude of any eccentric peak, if present (A_ecc_); the areas under the curve at eccentricities of 0.2° and 5°; and the half central peak radius. The latter was calculated for comparison with similar measures in previous studies^19^.

Computational modelling and initial model comparison was performed with *Mathematica*. Subsequent statistical analysis was performed using *Statistica* software (Statsoft Inc., 2300 East 14th St, Tulsa, Ok, 74104, USA). Data redundancy of derived parameters was investigated by factor analysis. Parameters of interest selected by factor analysis were then investigated further with multiple regression analysis between macular pigment-related parameters and morphometric parameters.

### Results 2: the M_3G_ model

#### Data fit and comparison of models

The mean sum of squares of the difference between the data and model (SSE) for the M_3G_ model was an order of magnitude smaller than that for the M_EG_ model for both the 2° and 5° data sets (Table 2). As with the M_EG_ model, M_3G_ parameters derived from the 2° data set could not be applied to model the 5° data set, which required a different parameter set for an optimum fit. The M_3G_ model fitted all data sets without manual parameter adjustments and did not exhibit the trade-off observed in the M_EG_ model between fitting the central peak and the peripheral tail-off.

The nine primary parameters of the M_3G_ model, determined from nonlinear regression of the 48 data sets with Eq. 2, are defined and presented in Table 3 together with the derived parameters. Parameter values for the model fitted to the data grand mean are presented in the final column of Table 3. The fit of the M_3G_ model to data grand mean, with its separate components, is presented in Fig. 2c, where it can be compared to the M_EG_ model (Fig. 2a, b). The first and second derivatives of the M_3G_ model fitted to the data grand mean together with points of interest dd_1_ - dd_6_ are presented in Fig. 2d.

**Table 3:** M_3G_ model primary and derived parameters.

	Definition	Fit to MPOD data 0° − 5°						Fit to data grand mean
		Mean	Min	Max	sd	95%CI	*n*	
Primary parameters								
N_1_	1st Gaussian component amplitude	0.224	0.100	0.447	0.102	0.195–0.253	48	0.200
N_2_	2nd Gaussian component amplitude	0.296	0.095	0.598	0.125	0.261–0.331	48	0.250
N_3_	3rd Gaussian component amplitude	0.097	0.015	0.382	0.092	0.071–0.123	48	0.039
p_1_	1st Gaussian component spread	32.092	2.044	180.000	43.978	19.650–44.533	48	14.515
p_2_	2nd Gaussian component spread	1.938	0.859	4.838	0.792	1.714–2.162	48	1.700
p_3_	3rd Gaussian component spread	-0.009	-0.098	0.070	0.032	-0.018–0.000	48	-0.020
g_1_	1st Gaussian component centre position	-0.027	-0.100	0.100	0.067	-0.046 - -0.008	48	-0.017
g_2_	2nd Gaussian component centre position	0.547	0.000	0.960	0.265	0.472–0.622	48	0.562
g_3_	3rd Gaussian component centre position	5.345	-2.000	10.000	5.243	3.861–6.828	48	10.000
Derived parameters								
A0_3G_	Central amplitude	0.650	0.245	1.169	0.201	0.593–0.707	48	0.649
A_ecc_	Amplitude of eccentric peak/convex inflection (dd4)	0.502	0.228	0.899	0.198	0.437–0.568	35	0.475
AUC02	Area under the curve 0° − 0.2°	0.125	0.044	0.230	0.041	0.113–0.136	48	0.125
AUC5	Area under the curve 0° − 5°	1.048	0.393	1.860	0.394	0.937–1.159	48	1.048
HHr	Radius of curve at half central amplitude (half height radius)	1.170	0.337	1.851	0.359	1.067–1.272	47	1.177
dd_1_	Eccentricity of 1st zero of second derivative	0.166	0.025	0.350	0.062	0.147–0.185	42	0.175
dd_2_	Eccentricity of 1st maximum of second derivative	0.283	0.009	0.750	0.116	0.249–0.318	43	0.296
dd_3_	Eccentricity of 2nd zero of second derivative	0.476	0.101	0.933	0.146	0.432–0.521	41	0.502
dd_4_	Eccentricity of 2nd minimum of second derivative	0.647	0.300	0.943	0.118	0.608–0.686	35	0.687
dd_5_	Eccentricity of 3rd zero of second derivative	1.117	0.474	1.574	0.218	1.051–1.183	42	1.086
dd_6_	Eccentricity of 2nd maximum of second derivative	1.457	0.878	2.009	0.267	1.382–1.533	48	1.497

#### Factor analysis

Factor analysis was performed on the three data sets – MPOD, OCT and OCTA (independent, X-variables) – along with the M_3G_ primary and derived parameters (dependent, Y-variables), to identify redundant variables that could be excluded in any subsequent analyses. Factor analysis using principal components^32^ identified which variables provided significant and independent information. Variables providing independent information were grouped into factors (F1, F2, etc.) that account for diminishing proportions of the total variance. Those variables providing significant information were quantified by a factor loading (FL). An absolute loading of |FL| > 0.7 was assumed to be significant. Detailed results are presented in the Supplementary Material and are summarised here. The analysis indicates a considerable degree of redundancy among variables, allowing for a reduced set of independent and dependent variables to be further analysed (Table 4).

Two MPOD X-variable factors accounted for 91% of the total variance (F1: 78%; F2 13%) with all variables except ODrad3 significantly loaded onto F1. No variable loaded significantly onto F2. This suggests that all variables loading onto F1 provide similar predictions for the dependent (Y) variables, with *sum V1* having the greatest absolute factor loading (-0.98). This result is expected, as all values tested are from the data set of measured and inter-related macular pigment variables.

Two factors accounted for 79% of the total OCT X-variable variance (F1: 47%; F2: 32%). Among the three significant F1 loadings, foveal bowl height had the greatest factor loading (bwlht, FL = 0.96) with foveal angle (fov ang, FL = -0.87) and foveal thickness (fov th, FL = -0.79) also being significant. For F2, average central retinal thickness (AvCRT, FL = -0.86) and foveal pit radius (Pr, FL = 0.85) had nearly equally loadings. Foveal bowl height (F1) and foveal pit radius (F2) were used in further analysis.

A single factor accounted for 97.6% of total OCTA/FAZ variable variance, with each variable (FAZ ferret, FAZ mean, FAZ equivalent radius) having loadings of approximately FL = -0.99. This indicates that any of the three variables would be sufficient as a dependent variable for subsequent analysis. FAZ equivalent radius (FAZer) was used in further analysis in keeping with data published elsewhere.

Five factors – F1 (28%), F2 (25%), F3 (20%), F4 (11%) and F5 (6%) – accounted for 90% of the total variance in the Y-variables (M_3G_ derived parameters), with F1 – F4 variable groupings having significant loadings (Supplementary Table S2). The variable with the greatest loading in each of the four groupings was used in further analysis (F1: dd_6_, FL = -0.83; F2: AUC02, FL = 0.87; F3: dd_4_, FL = -0.84; F4: p_1_, FL = 0.71). Notably, the F1 factor loading of half-height radius (HHr; FL = -0.74) ranked third, following dd_5_ (FL = -0.76); both were considered redundant and excluded from further analysis.

#### Multiple regression analysis

Multiple regression analysis was conducted between the X-variables and Y-variables selected through factor analysis. Two methods were used: (1) multiple regression (MR), which tested the significance of the regression slopes (β) and provided an equation expressing Y in relation to all tested X-variables^33^, and (2) stepwise multiple regression using the ‘forward’ method (SMR), which identified X-variables that were significantly related to Y and ranked them by importance^34^. A multiple regression coefficient of determination (R^2^) accounting for less than 50% of the total variance in Y was considered unlikely to be useful for prediction.

**Fig. 3:**
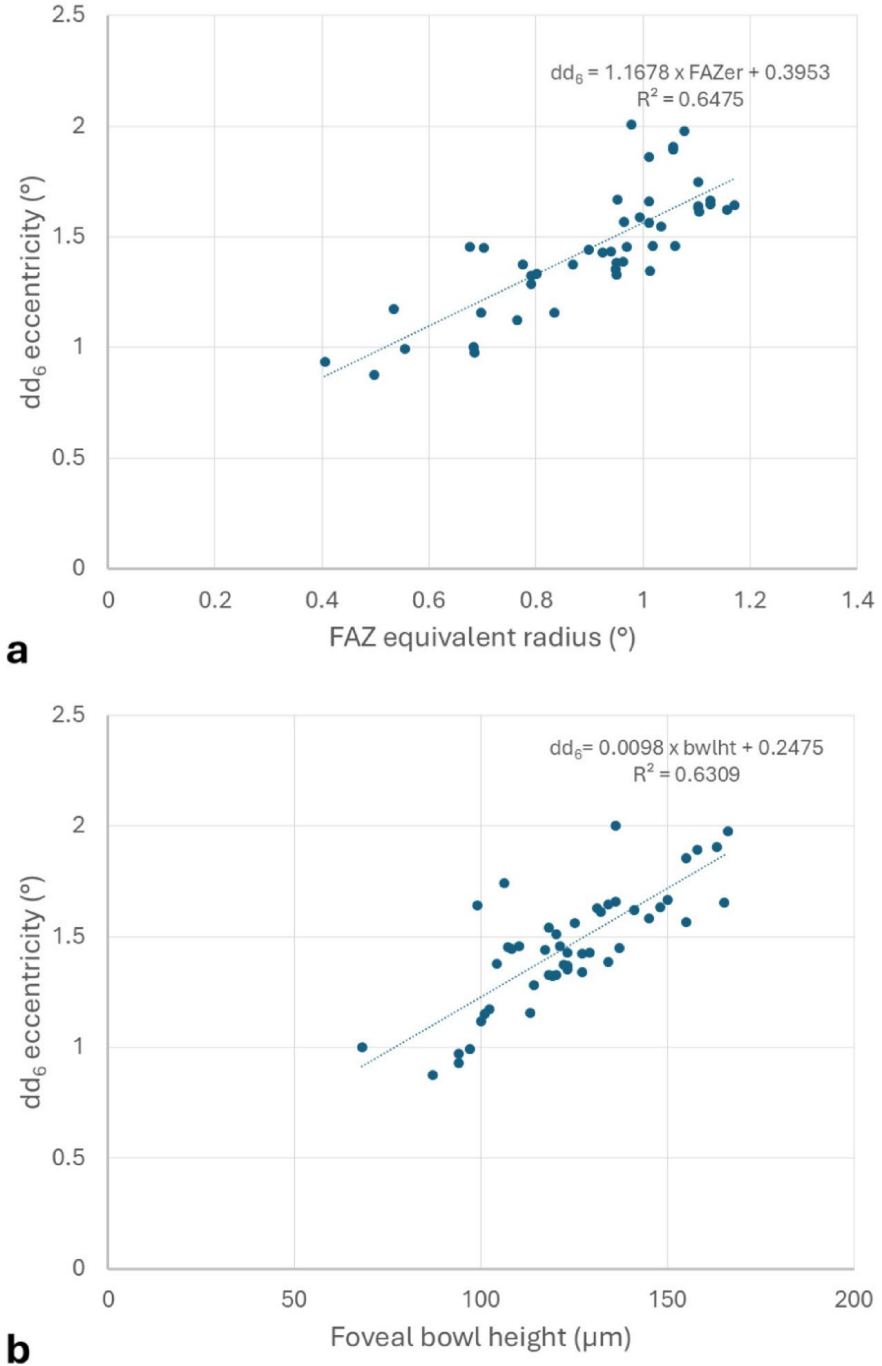
Relationship, with univariate linear regression model and coefficients of determination, between (a) foveal avascular zone equivalent radius (FAZer) and (b) foveal bowl height (bwlht) with M_3G_ derived parameter dd_6_.

Of all M_3G_ parameters selected by factor analysis (Table 4), dd_6_ had the greatest coefficient of determination (81%) being related to FAZ equivalent radius, foveal bowl height and foveal pit radius by the model: dd_6_ = 0.605*FAZer + 0.426*bwlht + 0.19*Pr. The univariate relationship between foveal avascular zone equivalent radius, foveal bowl height and dd_6_ is demonstrated in Fig. 3.

The three other selected M_3G_ variables combined accounted for less of the total M_3G_ variance than dd_6_ alone. The volume of macular pigment within the central 0.2° of eccentricity (AOC02, R^2^ = 24%) was significantly related to FAZ equivalent radius (β = -0.54) and foveal pit radius (Pr, β = 0.176). The spread of the first Gaussian component of M_3G_ (p_1_) was significantly related to foveal bowl height (bwlht, β = 0.237). No specific variables were significantly correlated with the eccentricity of eccentric peak/shoulder (dd_4_), although this parameter was selected by factor analysis into F3, which accounted for 20% of the M_3G_ parameter variance.

**Table 4:** Summary factor analysis with multiple regression and Stepwise multiple regression of selected dependent (Y) and independent (X) variables.

Factor analysis				Multiple regression			Stepwise multiple regression	
Grouping	% M_3G_ variance	Significant Y	Loading (FL)	Significant X	β	R^2^	Selected variables	R^2^ (%)
F1	28%	dd_6_	− 0.83	FAZer	0.603	81%	FAZer	65%
				bwlht	0.426		bwlht	13%
				Pr	0.190		Pr	3%
F2	25%	AUC02	0.87	FAZer	− 0.540	24%	FAZer	22%
				Pr	0.176			
F3	20%	dd_4_	− 0.84	None			None	
F4	11%	p_1_	0.71	bwlht	0.237	7%	bwlht	7%

## Discussion: interpretation

The initial aim of this study was to determine how an existing model of MPOD relates to experimental data of a wide range of MPOD spatial profiles. A further aim was to explore the biological and clinical significance, if any, of parameters derived from the model or its mathematical interrogation. The existing two-component exponential + Gaussian sum model provided a reasonable data fit for most data sets within the first 2° of eccentricity but was less effective for data sets extending to 5° eccentricity: fitting the model to account for shoulders and eccentric profile peaks was compatible with fitting either the central peak or peripheral tail-off but not both (Fig. 2a and b).

These preliminary findings led to the development of a novel three-component sum-of-Gaussians model, M_3G_, that provided a significantly better fit to more extended data sets, and MPOD profile details, than the best-fit version of the published model. The parameters of the first, second and third components of the M_3G_ model corresponded respectively to the central peak, any eccentric shoulder or eccentric peak, and the peripheral tail-off of the MPOD spatial profile (Fig. 2c).

The M_3G_ model was at least twice differentiable over assumed physiological ranges, allowing the shape of the M_3G_ curve to be interrogated, analysed and quantitatively compared with morphometric data. Whilst derivatives have been used in the analysis of macular pigment spectrophotometric data^35^, in macular spatial analysis^25,26^ and in fundus reflection spectroscopy^36^, such analysis appears not to have been performed on previously published macular pigment distribution models defined by continuous functions.

The first derivative of the M_3G_ function (M_3G_‘) describes the gradient of the M_3G_ curve, providing insight into the monotonicity and variability of the macular pigment optical density profile. The second derivative (M_3G_‘’) describes the rate of change this gradient with respect to eccentricity and is used to identify up to six critical points or points of interest (inflection and turning points) in the M_3G_ - modelled MPOD profile. For example, the radius of the fourth point of interest (dd_4_, the second minimum of the second derivative) precisely defines the eccentricity of the eccentric peak or shoulder of the profile. Areas under the M_3G_ curve are indices of macular pigment volume and can be obtained by integration between eccentricities of interest, such as from the centre (0°) to 0.2° and 5°.

Inspection of Figs. 2c and 3 shows that the MPOD spatial profile can be quantitatively separated into three sections by the first and second maxima of M_3G_’’. It is reasonable to hypothesise that these three sections correspond to the anatomical partitioning of the macular pigments. The central peak, between zero eccentricity and dd_2_, defined primarily by the first Gaussian component (i = 1) of M_3G_, relates to the central MPOD peak/Mueller cell cone/ zeaxanthin/mesozeaxanthin locus. The intermediate section, between dd_2_ and dd_6_, defined primarily by the second Gaussian component (i = 2) of M_3G_, incorporating any shoulder or eccentric peak, relates to the more eccentric IPL/OPL/Henle fibre/zeaxanthin/mesozeaxanthin locus: the eccentricities of shoulders, secondary peaks being quantified by the eccentricity of the local minimum of M_3G_’’ between these values (dd_4_). The third section from dd_6_ to the extent of measurable retina, defined primarily by the third Gaussian component (i = 3) of M_3G_, relates to the low background lutein-binding layers and centrifugal tail-off of macular pigment optical density.

This hypothesis requires further study using high-resolution morphometry, particularly of the Henle fibre layer. The Henle fibre layer is difficult to image quantitatively with available instrumentation and was not measured in the present study. Quantification of the Henle fibre layer, for example, using polarization sensitive OCT^37^ or OCT methods based on the manual misalignment of OCT instrumentation^38,39^, could provide valuable insights into the relationship between this layer and macular pigment distribution, the foveal avascular zone and foveal morphology in general.

**Fig. 4:**
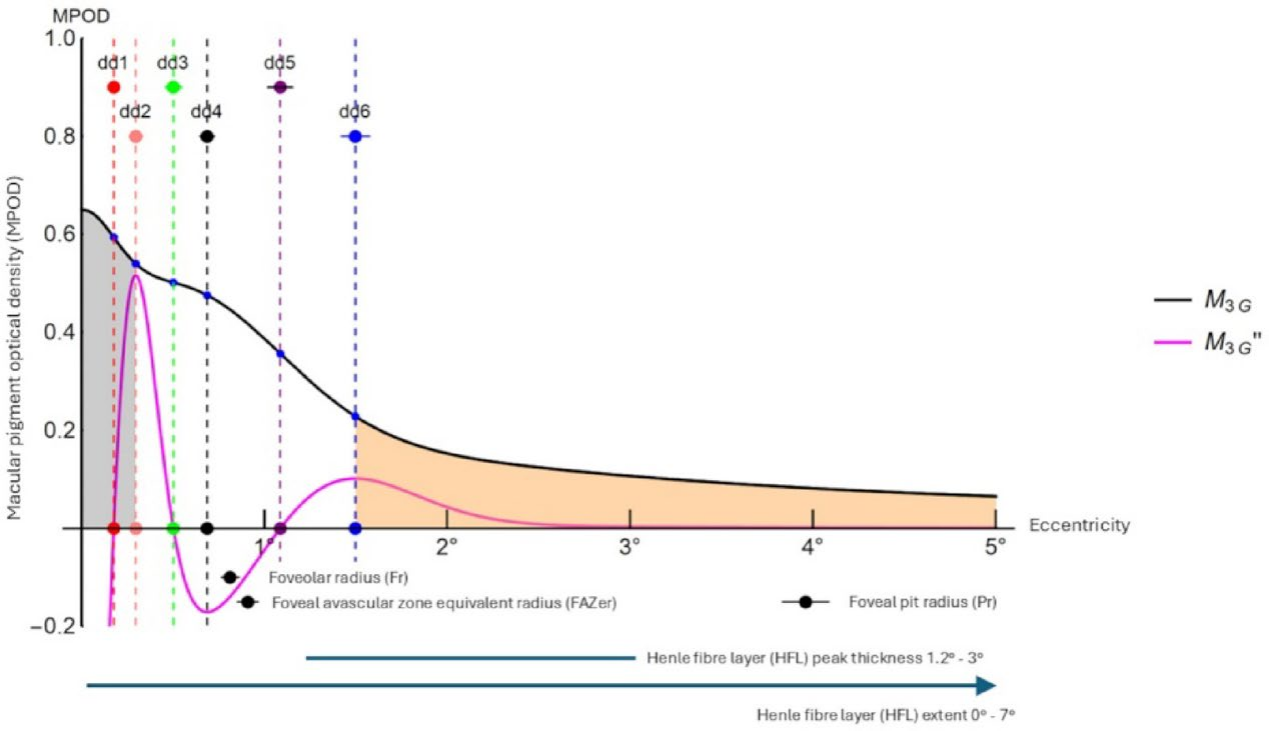
Summary graph showing the M_3G_ model fitted to the grand mean MPOD data (black continuous curve) and its second derivative (M_3G_’’, magenta continuous curve) compared to structural landmarks (black annotations below x-axis). Eccentricities of points of interest dd_1_ – dd_6_ are identified by coloured dots and broken vertical lines. The grey shading is the area under the M_3G_ curve between the centre point (eccentricity = 0°) and the eccentricity of the first local maximum of M_3G_’’ (dd_2_ = 0.28°); the orange shading is the area under the M_3G_ curve from the eccentricity of the second local maximum of M_3G_’’ (dd_6_ = 1.46°) to the eccentricity limit of the graph (5°). Horizontal data bars are 95% confidence intervals.

Data fitting of the M_3G_ model to individual eyes allowed extraction of variables for correlation with morphometric data. The relationship of the M_3G_ curve and its specific features with key macular morphological landmarks is shown in Fig. 4. The greatest proportion of macular pigment lies within the first half of the radius of the foveal pit and central to the peak thickness of the Henle fibre layer, which extends beyond the 5° boundary of the graph. The inflection point of any MPOD eccentric peak or shoulder, identified by the eccentricity of the local minimum of M_3G_’’ (dd_4_), is located just within the boundary of the foveal avascular zone and foveolar radius.

Quantitative assessment of the relationship between the distribution of macular pigment and morphology was preceded by factor analysis, which demonstrated significant data redundancy in both M_3G_ variables and morphometric variables. The variable with greatest factor loading in each factor was used in this study to focus subsequent multiple regression analysis. Significant variables within each factor with lesser loadings are not invalidated by the factor analysis and might benefit from further investigation.

Multiple regression analysis of the selected variables demonstrated a strong correlation between the eccentricity of the second maximum of M_3G_ ‘’ (dd_6_) and both foveal avascular zone equivalent radius and foveal bowl height. The dd_6_ variable is a constant feature in all eyes examined and may be taken as a definable boundary of the radial extent of macular pigment. In other words, the area of macular pigment correlates with the depth of the foveal pit and the area of the foveal avascular zone. This conclusion is compatible with known association between larger foveal avascular zones and deeper, broader foveal pits^40^.

Furthermore, the positive correlation between the radial extent of macular pigment and the foveal avascular zone equivalent radius is consistent with the positive correlation between the latter and the eccentricity of the half-height of the peak central macular pigment density (half-height radius)^19^. Whilst the half-height radius metric is mathematically meaningful as an index of the ‘spread’ of macular pigment if the profile were purely exponentially declining (being analogous to the half-life constant of exponential decay), it is less meaningful in a non-exponential decline and is potentially ambiguous in a non-monotonic decline, particularly where there is a shoulder or eccentric peak with values near to the half-central peak height value.

There is no such ambiguity with the eccentricities of either the primary or derived parameters of the M_3G_ model, and the strong structural correlations of the dd_6_ eccentricity support their value in defining the radial extent of macular pigment. Furthermore, the area under the MPOD profile curve from zero to an eccentric point of interest, such as dd_6_, might be a clearly defined and useful individualised metric of total macular pigment volume.

Other potentially useful metrics of macular pigment highlighted by the factor analysis include the central macular pigment volume, quantified by AOC02, and the radius of the eccentric peak/shoulder, quantified by dd_4_. Amplitude values of M_3G_ at the dd_4_ and dd_6_ eccentricities, whilst not investigated in the present study, require further evaluation, as do their clinical applications. Additional metrics of interest can be derived from the M_3G_ model depending on the intended application. For example, the eccentricity at which the volume of macular pigment is 50% (or another fraction) of a predefined limiting eccentricity can be readily calculated.

The precise fit of the M_3G_ model when applied to experimental data has potential clinical utility in data interpretation and analysis. The function can be used to effectively smooth otherwise noisy clinical data, with the primary and derived parameters providing potentially clinically useful information of macular pigment densities, volumes and the shape of the macular pigment distribution in the retina.

In summary, this study demonstrates that a new model of the macular pigment optical density profile accurately represents measured data to retinal eccentricities of 5° and beyond. Useful and consistent data can be obtained particularly from second derivative critical points, which correlate with OCT-derived morphological features. Whilst there is redundancy between variables, factor analysis identified four parameters that strongly correlate with morphometric landmarks, particularly the extent of the foveal avascular zone. The macular pigment density profile, as modelled by M_3G_, can be subdivided naturally into three components, which, it is hypothesised, correspond to the observed anatomical segregation of the macular pigments.

A sum of three Gaussian model is flexible in that it can, with careful parameter selection, be used to define a large variety of different profiles. It is noteworthy that a similar model has been used to accurately represent central macular retinal thickness and topography^25,26^. The similarity of these models suggests a possible integration of macular pigment and structural models, and raises a deeper question as to what common processes, if any, are involved in the formation of macular topography and the deposition of macular pigment.

## Supplementary Information

Below is the link to the electronic supplementary material.

Supplementary Material 1

## Data Availability

The datasets generated during and/or analysed during the current study are available from the corresponding author on reasonable request. Further detailed analysis is available in the Supplementary Information.
