# Supplementary material for: A novel computational model for human macular pigment optical density and its relationship to foveal structure
Source: Sci Rep. 2025 Oct 29;15:37865. doi: 10.1038/s41598-025-21681-4 (PMC12572246; doi:10.1038/s41598-025-21681-4)
Supplement: Supplementary file 1 — Supplementary Material 1 [file 41598_2025_21681_MOESM1_ESM.pdf]

A Novel Computational Model for Human Macular Pigment Optical Density and Its Relationship to Foveal Structure

Supplementary Material

Gary P Misson[a, b] * (https://orcid.org/0000-0001-8843-8389),

Stephen J Anderson[a] (http://orcid.org/0000-0002-5719-2846)

Richard A Armstrong [a] (https://orcid.org/0000-0002-5046-3199)

Rebekka Heitmar [c] (https://orcid.org/0000-0002-7657-1788)

[a] School of Optometry, College of Health and Life Sciences, Aston University, Birmingham, UK.

[b] South Warwickshire NHS Foundation Trust, Lakin Road, Warwick, UK

[c] Centre for Vision across the Life Span (CVLS), School of Applied Sciences,, University of Huddersfield, UK.

*Corresponding author

Table S1 Model mean parameter values for optimised data fits of M_{EG} and M_{3G} models to data ranges 0° - 2°and 0° - 5°. Significant differences ($p < 0.05$) between pairs for each model are marked with *.

M_{EG}						M_{3G}				
Data range:		0° - 2°	0° - 5°					0° - 2°	0° - 5°	
A_1	mean	0.5813	0.4406	*		N_1	mean	0.2504	0.2464	
	sd	0.2066	0.1601				sd	0.1485	0.1121	
A_2	mean	0.1448	0.2226	*		N_2	mean	0.2778	0.3466	*
	sd	0.1291	0.1639				sd	0.1328	0.1606	
						N_3	mean	0.1781	0.1781	
							sd	0.1414	0.1414	
p_1	mean	0.6862	0.5057	*		q_1	mean	28.18	37.83	
	sd	0.2431	0.2065				sd	40.93	54.23	
p_2	mean	5.7742	4.5571			q_2	mean	2.012	1.633	*
	sd	3.8938	4.3094				sd	0.8024	1.016	
						q_3	mean	0.06183	0.06612	
							sd	0.2432	0.1523	
						g_1	mean	0.02553	-0.04239	
							sd	0.06436	0.06210	
x_2	mean	0.8875	0.5983	*		g_2	mean	0.5596	0.4941	
	sd	0.4323	0.3971				sd	0.2656	0.2868	
						g_2	mean	2.517	2.3259	
							sd	4.205	3.456	

Definitions of derived parameters

For either function M = M_{EG} or M_{3G}, the following can be defined:

1) Areas under the curve for given eccentricity c

$$AOC_M(c) = \int_0^c M.dx$$

For this study, c = 0.2° and c = 5.0° [$AOC_M(0.2)$, $AOC_M(5.0)$].

2) The half peak radius, RHP_M, is obtained by numeric solution of:

$$\mathrm{M}(RHP_M) = \frac{\mathrm{A0_M}}{2}$$

Statistical Analysis

All analyses were carried out using STATISTICA software (Statsoft Inc., 2300 East 14th St, Tulsa, Ok, 74104, USA).

The Independent, X-variable data sets were MPOD, OCT and OCTA. The dependent, Y-variables data sets were M_{3G} primary and derived parameters.

Factor analysis (FA) was applied to the Y variables and each set of X variables to determine the degree to which variables within a set were confounded. Analysis used principal components (PC) as the extraction method and initial solutions were unrotated (Hilton and Armstrong 2011). The output from such an analysis is a series of eigenvalues ('latent roots') which are proportional to the variation accounted for by each axis, the eigenvectors ('latent vectors') representing the 'loadings' (Factor Loading, FL), i.e., the spatial co-ordinates of each variable in relation to the factors. Several factors are extracted from the data each accounting for a specific proportion of the total variance, F1 for the greatest individual proportion of the variance and remaining factors (F2, F3… etc) for diminishing amounts of the remaining variance. Normally, two or three factors account for most of the variance within the data, the fourth and successive factors accounting for small and diminishing amounts of the residual variation, unless considerable heterogeneity is present.

To obtain the simplest model describing the relationship between the Y and X variables, a multiple regression analysis was carried out based on the results of the FA.

Y variables with greatest loading from each of the relevant factors (F1, F2 etc) for each data set was tested against the three groups of X variables using: (1) multiple regression (MR) which tested the significance of the regression slopes (β) and provides an equation expressing Y in relation to the X variables (Hilton and Armstrong 2011)and (2) stepwise multiple regression using the 'forward' method (SMR) which identified which X variables are significantly related to Y in order of their importance (Hilton and Armstrong 2011).

Factor Analysis

1. Y variables: M_{3G} primary and derived parameters

(a) Five 'factors' were extracted accounting for 90% of the total variance: F1 (28%), F2 (25%), F3 (20%), F4 (11%) and F5 (6%) in diminishing order of importance.

(b) Factor loadings of the Y variables on these factors are shown in Table S2. Significant loadings (those with loadings > 0.7 are in bold.

Table S2 Y-variable Factor Analysis

Variable	Factor Loading				
Y	F1	F2	F3	F4	F5
A1	-0.5194	0.2413	-0.3476	-0.5124	0.1994
A2	-0.5414	0.5101	0.6063	-0.0827	-0.0523
A3	-0.7999	-0.2273	0.3564	0.0858	-0.1889
p1	0.1977	-0.2785	0.2618	**0.7100**	-0.3682
p2	0.5139	0.1845	-0.1872	0.5696	0.4857
p3	**-0.7250**	-0.4166	0.2062	-0.1680	-0.1594
x1	0.4073	0.5539	-0.3098	0.3542	-0.4871
x2	-0.4584	-0.0448	**-0.7455**	0.4094	0.1966
x3	0.6207	0.5529	-0.2668	0.1643	0.1942
A0	-0.3947	**0.8147**	0.3226	-0.0389	0.1703
Aecc	-0.4776	**0.7280**	0.3937	0.2672	0.0980
A0C02	-0.3590	**0.8683**	0.2924	0.0176	0.1056
A0C5	-0.6007	0.6794	0.2415	0.2901	0.0706
HH	**-0.7395**	-0.1704	-0.2400	0.5617	-0.0607
dd1	0.1887	**0.7628**	-0.4093	-0.1009	-0.4170
dd2	0.0685	**0.7153**	-0.3014	-0.1745	-0.2910
dd3	-0.0033	0.3452	**-0.7780**	-0.4361	-0.0386
dd4	-0.3163	0.1527	**-0.8383**	0.1847	0.0433
dd5	**-0.7615**	-0.2264	-0.5784	0.1328	0.0171
dd6	**-0.8355**	-0.2823	-0.4242	-0.0498	-0.0890
% total variance:	28%	25%	20%	11%	6%

(c) Four groupings of variables are evident (F5 has no significant loadings)

F1: *p3, half height, dd5, dd6* (*dd5* and *dd6* have 'best' loadings)

F2: *A0, Aecc, A0C02, dd1, dd2* (*A0C02* best)

F3: *x2, dd3,dd4*, (*dd4* best)

(d) Fig S1 displays the factor loading in relation to F1 and F2and illustrates the close relationship between the variables loaded on F1.

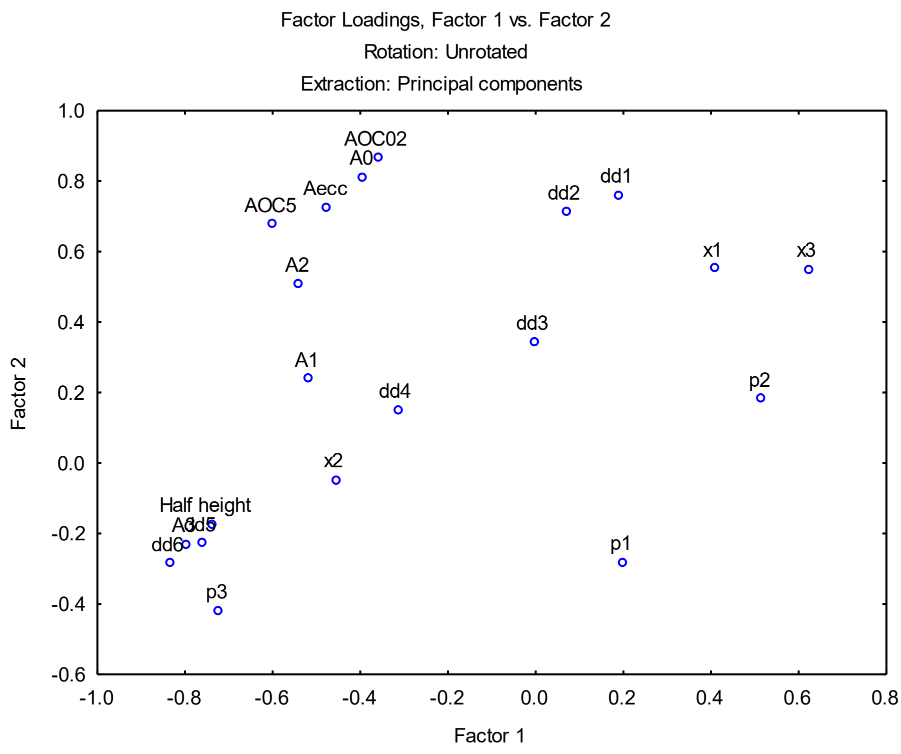

Fig S1. Factor analysis of the Y (dependent, outcome) variables: A plot of loadings in relation to F1 and F2

2. X variables: MPOD

(a) Two factors extracted accounting for 91% of the total variance (F1 (78%), F2 (13%)

(b) Factor loadings of the MPOD variables are shown in Table S3.

Table S3 X-variable Factor Analysis: MPOD

Variable	Factor Loading	
MPOD	F1	F2
OD rad0.2	**-0.8849**	0.4548
OD rad 1	**-0.9285**	-0.2772
OD rad 2	**-0.8850**	-0.4030
OD rad 3	-0.6518	-0.2800
sumV0.2	**-0.8682**	0.4908
sumV1	**-0.9791**	0.0230
sumV2	**-0.9505**	-0.2529
sumV3	**-0.9413**	-0.2758
peak	**-0.8356**	0.5331
% total variance:	78%	13%

(c) There is a single group of confounded variables loading significantly on F1, i.e. all MPOD variables except *ODrad3*. No variables are significantly loaded on F2.
(d) All variables loaded on F1 provide similar predictions of Y, *sumV1* being 'best'.

(e) Fig S2 displays the factor loading in relation to F1 and F2

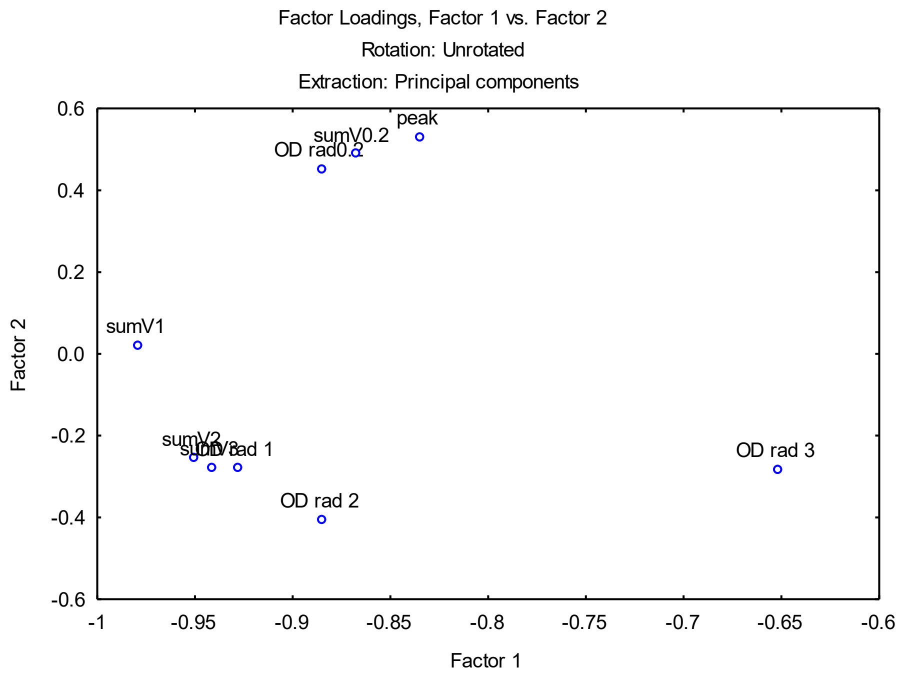

Fig S2. Factor analysis of the X (independent, input) MPOD variables: A plot of loadings in relation to F1 and F2

3. X variables: OCT

(a) Two factors were extracted accounting for 79% of the total variance (F1 = 47%, F2 = 32%)

(b) Factor loadings of the OCT variables are shown in Table S4.

Table S4 X-variable Factor Analysis: OCT

Variable OCT	Factor Loading F1	F2
Av CRT	0.0755	**-0.8563**
fov th	**-0.7913**	-0.3916
bwl ht	**0.9573**	-0.0564
fov ang	**-0.8747**	0.4358
Pr	-0.0230	**0.8481**
Fr	0.6916	0.3030
% total variance:	47%	32%

(c) Two groups of variables:

F1: *fov th, bwl ht, fov ang* (*bwl ht* 'best')

F2: *AvCRT, Pr* (both very similar loadings)

Bwl ht and one of *AvCRT* or *Pr* provide independent information of the X variables

(d) 2D display (fig 3) not especially revealing with all variables more or less scattered in the space

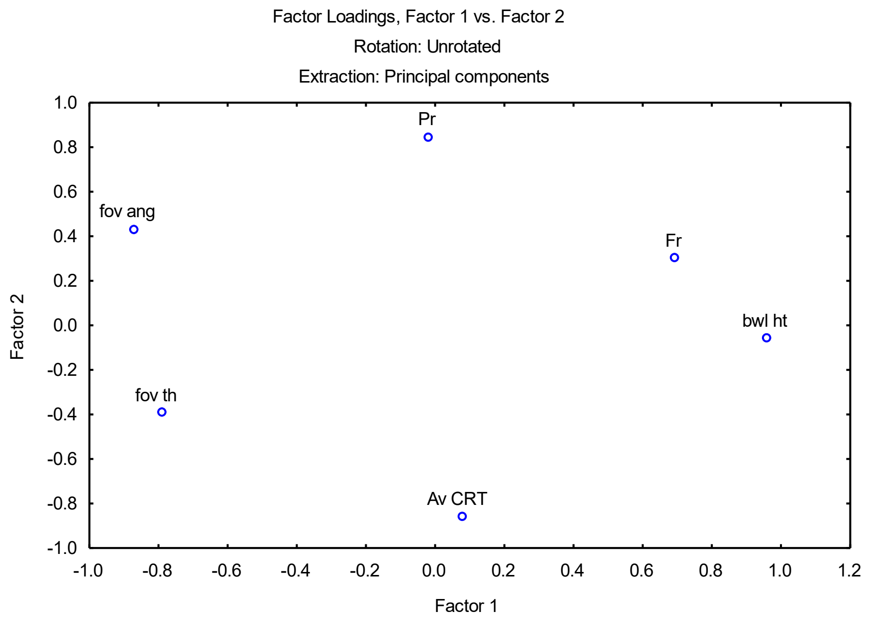

Fig 3. Factor analysis of the X (independent, input) OCT variables: A plot of loadings in relation to F1 and F2

4. X variables: OCTA/FAZ

(a) one factor (F1) extracted accounting for 97.6% of total variance.

(b) Factor loading are shown in Table S5

Table S5 X-variable Factor Analysis: OCT

Variable OCTA/FAZ	Factor loading F1
FAZ Feret	**-0.9850**
FAZ(W+H)/2	**-0.9916**
Eq radius	**-0.9879**
% total variance:	97.60%

(c) All three variables have high loadings on F1 and are very similar

(d) Any single variable would be sufficient as a suitable dependent variable

(e) No plot possible as only one significant factor extracted.

5. Factor Analysis: Overall conclusions

(a) The analyses indicate which variables provide independent information (those loaded onto different factors) and those variables providing significant information (those with significant loadings on a factor, the higher the loading indicates greater significance). The analysis suggests a considerable degree of redundancy among these variables.

(b) The analysis suggests that there are four groups of Y variables in diminishing order of importance (F1 being the most important). The variables within each group are likely to be very similar in their responses. Most important independent outcome variables would be those with the highest loading within the group in diminishing order of importance *dd6* (F1), *AOCO2* (F2), *dd4* (F3), *p1* (F4).

(c) All the MPOD variables are loaded on F1 and provide similar predictions of Y, *sumV1* being 'best'. However, the loadings of all these variables are very similar and any of them or any combination of them likely to be a good predictor. This is to be expected and confirms given that all the MPOD variables represent aspects of the MPOD distribution.

(d) The analysis of the OCT variables extracts two groups of independent variables, *bwl* or one of *AvCRT* and *Pr* are likely to be a good predictor. As the foveal bowl height and average central retinal thickness measure similar features of the central fovea, foveal pit radius was (Pr) was chosen for regression analysis.

(e) All three OCTA/FAZ variables show high loadings on F1. They are very similar predictors of Y and any one of them alone or in combination could be used. Foveal avascular zone equivalent radius (FAZer) was chosen for further analysis in keeping with previous studies (Balaratnasingam, Chae et al. 2015).

Multiple regression (MR) and stepwise multiple regression (SMR) analyses of selected Y and X variables based on FA

1. *Objective*: Based on the results of the FA, to test a series of four dependent (Y) variables, *p1, AOCO2, dd4, dd6* against three X variables: *bwl ht, Pr, eq radius*.

2. Results (Table S6). MR & SMR analysis reduces to a simple message: the best predicted outcome (Y) is *dd6* using *eq radius* ,*bwl ht* and *Pr* as predictors. All other variables are redundant, not significant, or not well predicted.

'Best' model is: *dd6* = 0.605*eq radius* + 0.426*bwl ht* + 0.19*Pr*

Table S6. Summary of multiple regression (MR) and stepwise multiple regression (SMR) analyses of FA-selected variables

FA		MR			SMR	
Y	FA grouping	R^2	X	β	Selected variables	R^2 (%)
dd6	F1	81%	eq radius	0.603	eq radius (65%)	65%
			bwl ht	0.426	bwl ht	13%
			Pr	0.19	Pr	3%
AOCO2	F2	24%	eq radius	-0.54	eq radius	22%
			Pr	0.176		
dd4	F3	5%	None		None	
p1	F4	7%	bwl ht	0.237	bwl ht	7%

Supplementary Material References

Balaratnasingam, C., B. Chae, M. H. Remmer, E. Gomez, M. Suzuki, M. Engelbert and R. F. Spaide (2015). "The Spatial Profile of Macular Pigments Is Related to the Topological Characteristics of the Foveal Avascular Zone." Investigative Ophthalmology & Visual Science **56**(13): 7859-7865.

Hilton, A. and R. A. Armstrong (2011). "Statnote 24: Multiple regression." Microbiologist **12**(1): 40 - 43.

Hilton, A. and R. A. Armstrong (2011). "Statnote 25: Stepwise multiple regression." Microbiologist **12**(2): 38 - 39.

Hilton, A. and R. A. Armstrong (2011). "Statnote 27: Principal components analysis (PCA)." Microbiologist **12**(4): 37 - 40.
